# Race-, Age-, and Anatomic Site-Specific Gender Differences in Cutaneous Melanoma Suggest Differential Mechanisms of Early- and Late-Onset Melanoma

**DOI:** 10.3390/ijerph16060908

**Published:** 2019-03-13

**Authors:** Tze-An Yuan, Yunxia Lu, Karen Edwards, James Jakowatz, Frank L. Meyskens, Feng Liu-Smith

**Affiliations:** 1 Program in Public Health, University of California Irvine, Irvine, CA 92697, USA; tzeany@uci.edu (T.-A.Y.); yunxial1@uci.edu (Y.L.); kedward1@uci.edu (K.E.); flmeyske@uci.edu (F.L.M.); 2 Chao Family Comprehensive Cancer Center, University of California Irvine, Irvine, CA 92697, USA; 3 Department of Epidemiology, School of Medicine, University of California Irvine, Irvine, CA 92697, USA; 4 Department of Surgery, University of California Irvine, Irvine, CA 92697, USA; jgjakowa@uci.edu; 5 Melanoma Center, University of California Irvine, Irvine, CA 92697, USA; 6 Department of Medicine, School of Medicine, University of California Irvine, Irvine, CA 92697, USA

**Keywords:** melanoma, gender difference, race difference, incidence rate, incidence rate ratio, anatomic body site, UV, non-white, annual average percentage change

## Abstract

In order to explore melanoma risk factors through gender-, age-, race-, and site-specific incidence rates, malignant melanoma cases from the Caucasian whites and non-whites were retrieved from the US SEER database. Age-standardized, age-, and site-specific tumor rates were calculated. All races and both genders showed positive annual average percentage changes (AAPCs) over the years, but AAPCs varied at different body sites, with men’s trunk exhibiting the fastest increase. Non-whites were diagnosed at a significantly younger age than whites and showed a trend towards fewer gender differences in the age of diagnosis. However, non-whites and whites showed a similar pattern of age-specific gender differences in the incidence rate ratios. A consistent spiked difference (female vs. male, incidence rate ratio (IRR) >2) was observed at or near the age of 20–24 in all race groups and at all body sites. The highest female vs. male IRR was found in the hip and lower extremities, and the lowest IRR was found in the head and neck region in all races. These race-, gender-, and site-dependent differences suggest that age-associated cumulative sun exposure weighs significantly more in late-onset melanomas, while genetics and/or pathophysiological factors make important contributions to early-onset melanomas.

## 1. Introduction

Malignant cutaneous melanoma (CM) is the number one cause of death in skin cancer patients in the US [1], and the incidence rate has continued to increase since the 1930s [2]. The risk factors have not been completely elucidated, but a pathophysiological role of sex has been suggested in addition to UV radiation [3,4,5]. The sex differences in body site distribution of CM incidence rates have contributed to this assumption [6,7,8,9]. Of the sex differences, men have tumors predominantly on the trunk, especially on the back [10], while women have more tumors on the lower extremities [10]. In recent years, CMs in the head and neck area are rising in men, with subtle distinctions identified between sexes on the face [11]. UV radiation has long been considered the primary environmental risk factor for CM because of UV-induced DNA damages and mutations [12,13,14]. The distribution of CM on the body surface has been recognized to reflect UV impact as different body sites receive different levels of UV exposure [15,16]. To date, the main hypothesis to explain the bodily distributive differences in CM lies heavily in UV exposure and its associated behavioral factors, such as clothing styles and use of cosmetics and occupation choice, which may reflect differences between genders [11,17].

Additional melanoma risk factors to UV exposure have been suggested by recent epidemiological publications [4,18]. It had been reported since 1975 that women under the age of 50 showed higher incidence rates than men in the same age range [19]. The underlying etiological mechanisms have been largely attributed to UV exposure, including the impact of ambient UV radiation and indoor tanning popularity in younger women [20]. Nevertheless, this sex discrepancy of UV exposure did not result in a similar age-specific incidence difference in non-melanoma skin cancers (NMSCs) [4], which are mostly found in sun-exposed body sites [15,21] and exhibit a straightforward causative relationship with UV exposure [22]. In support of a less straightforward role of UV exposure, melanoma is frequently found in less sun-exposed body areas (i.e., trunk) [23]. Moreover, increased sunscreen use has not shown a preventive role in melanoma incidence rates in sun-exposed body areas [24], which raised a question as to whether the current UV-based primary prevention message was sound [25].

The goal of the current study was to understand melanoma disparity and differential etiology among different ages, genders, and race groups through analyzing melanoma tumor distributions in four major body regions—the head and neck region, the shoulder and upper extremities, the trunk, and the hip and lower extremities, using US Surveillance, Epidemiology, and End Results Program (SEER) data. These results suggested that melanomas diagnosed at younger and older ages may show different risk profiles which warrant further investigation.

## 2. Materials and Methods

### 2.1. Study Design

This is a retrospective cancer registry-based cohort analysis using US Surveillance, Epidemiology, and End Results Program (SEER) data.

### 2.2. Study Population

For body-site specific incidence rate analysis, the SEER18 database (Incidence—SEER18 Regs Research Data + Hurricane Katrina Impacted Louisiana Cases, Nov 2017 Sub (2000–2015) <Katrina/Rita Population Adjustment>) was downloaded through SEER*Stat software (version 8.3.5). Melanoma cases were collected from 18 SEER registries, including San Francisco–Oakland SMSA, Connecticut, Detroit (Metropolitan), Hawaii, Iowa, New Mexico, Seattle (Puget Sound), Utah, Atlanta (Metropolitan), San Jose–Monterey, Los Angeles, Alaska Natives, Rural Georgia, California (excluding SF/SJM/LA), Kentucky, Louisiana, New Jersey, and Greater Georgia. Caucasian white melanoma cases (*n* = 262,130) were retrieved in this study, including 152,666 men and 109,464 women. Non-white patient cases, including black Americans, Hispanics, American Indians/Alaska Natives, and Asian Americans/Pacific Islanders (*n* = 11,295), of 5088 men and 6207 women were also retrieved to make comparisons with the white population. For calculating the annual percentage change of incidence rates, age- and sex-specific rates for each body site were downloaded similarly via SEER*Stat software and adjusted by the US 2000 standard population. For the incidence trend analysis, SEER data from 1973–2015 was used.

### 2.3. Definition of Melanoma

Melanoma was defined based on the ICD-O-3/WHO 2008 site recode as “Melanoma of the skin” and having AYA site recode/WHO 2008 category of “7.1 Melanoma”. ICD-O-3/WHO 2008 primary site code “C44-Skin” includes “C44.0-Skin of lip (NOS, not otherwise specified), C44.1-Eyelid, C44.2-External ear, C44.3-Skin of other and unspecified parts of face, C44.4-Skin of scalp and neck, C44.5-Skin of trunk, C44.6-Skin of upper limb and shoulder, C44.7-Skin of lower limb and hip, C44.8-Overlapping lesion of skin (overlapping with other malignant neoplasms of skin), and C44.9-Skin (NOS, not otherwise specified)”, which were categorized into 4 groups in the present study as follows: C44.0—C44.4 as “Head/Neck” region, C44.5 as “Trunk” region, C44.6 as “Upper” region, and C44.7 as “Lower” region. The C44.8 and C44.9 categories were showing a zero number of cases in the database. ICD-O-3/WHO 2008 Hist/behave, malignant categories (excluding melanoma in situ) of melanoma “8720/3-8723/3, 8726/3, 8727/3, 8730/3, 8740/3-8746/3, 8761/3, 8770/3-8774/3, and 8780/3” were included in the present study.

### 2.4. Variables

The outcome of interest in the current study was the incidence rate of melanoma (definition above). We analyzed the incidence rates separately by the four major anatomic sites as abovementioned. Age (0–4, 5–9, …, 80–84, 85+, and all ages combined), gender (female and male), and race (Caucasian white and non-white combined) were the major exposures of interest. No other covariates were used in the current study in addition to these primary variables of interest.

### 2.5. Statistics

Age-specific incidence rates were calculated by case number divided by population (per 100,000 person-years) and presented in 19 age groups (0–4, 5–9, …, 80–84, 85+, and all ages combined). Age-standardized rates were adjusted to the US 2000 standard population. The rate ratios were calculated using female incidence rates divided by male rates as previously described [26]. The 95% confidence intervals of the rate ratios were calculated by Stata (version 13.1, StataCorp LLC, College Station, Texas, USA) [27]. The incidence rates were stratified by the four major anatomic sites, two genders, and 19 age groups as crude unadjusted results without controlling for potential confounders. Incidence rates in different race groups were presented in separate tables. The average annual percentage changes of incidence rates were produced by the SEER Joinpoint Regression [28] Program (version 4.6.0), downloaded from the SEER website based on SEER*Stat data from 1973–2015 (age-adjusted). The mean diagnosed age comparison was made by the two-sample student t-test with no correction for multiple comparisons. The two-sided significance level was set at 0.05 by default.

## 3. Results

### 3.1. Patient Characteristics and Differences in the Mean Age of Diagnosis

Caucasian white (“white” in short) melanoma cases (*n* = 262,130) in the SEER18 database (2000–2015) were retrieved, including 109,464 (41.8%) women and 152,666 (58.2%) men (Table 1). Melanoma cases from the non-white races including black Americans, non-white Hispanics, American Indians/Alaska Natives, and Asian Americans/Pacific Islanders (SEER18 database, 2000–2015) were also retrieved (Table 1, total 5088 men and 6207 women). Case distribution in the head and neck region (“Head/Neck”), the upper limbs and shoulder (“Upper” in short), the trunk, and the lower limbs and hip region (“Lower”) in each gender and race is listed in Table 1. The total white cases were 262,130, and the non-white cases were 11,295. The percentage of women was higher (55.0%) in the non-white populations but lower in the white population (41.8%). Overall, men were diagnosed at older ages than women in all body sites and in all race groups (Table 1). The mean age of diagnosis in the Head/Neck region was the oldest among all body sites, also in all race groups. The youngest mean age of diagnosis varied in both genders and all race groups (Table 1). While the mean age of diagnosis for all sites did not show a significant gender difference in the white population (*p* = 0.18, two-sample *t*-test), the mean diagnosis ages in the Head/Neck, Trunk, and Lower areas were significantly different between white men and white women. Only the Upper region did not show a significant gender difference (*p* = 0.51). In the non-white populations, however, there was no significant gender difference in any body site or in all body sites combined. The mean age of diagnosis was significantly younger in the non-white groups than the whites in both genders.

### 3.2. The Age-Specific Melanoma Incidence Rates in Each Body Site in the White Population

**Figure 1:**
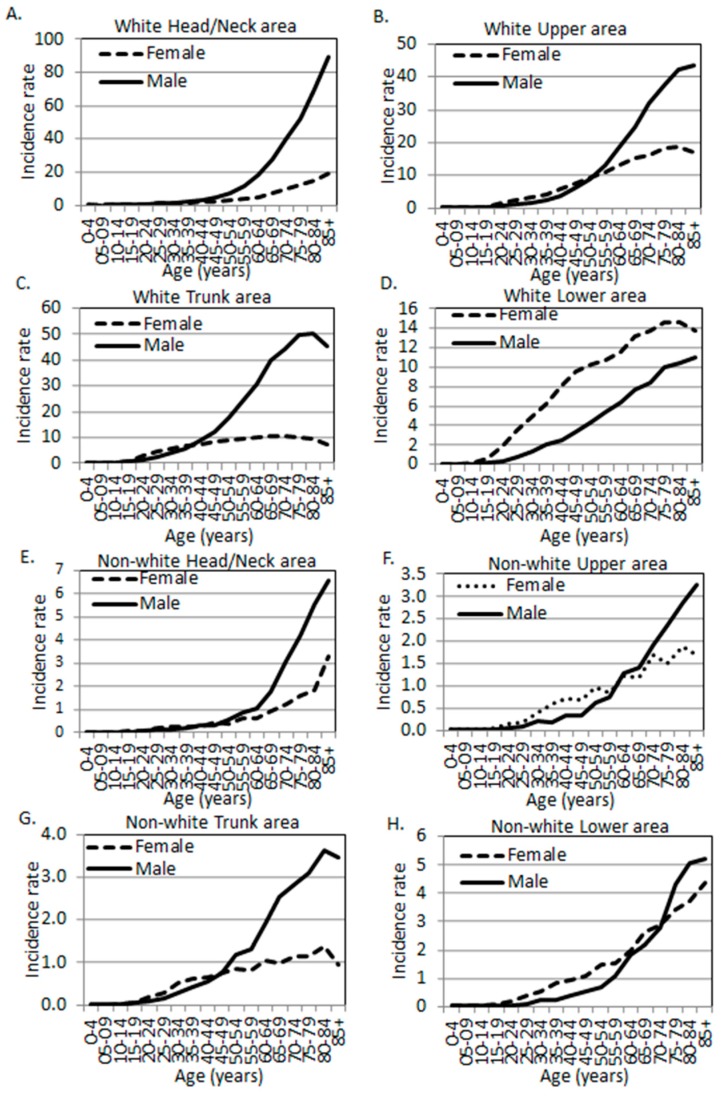
Melanoma incidence rates by age, sex, and anatomic site. (**A**–**D**) White population. (**E**–**H**) Non-white populations. (**A**,**E**) Head and neck area. (**B**,**F**) Upper limbs and shoulder area. (**C**,**G**) Trunk. (**D**,**H**) Lower limbs and hip area (SEER 18 data, 2000–2015).

The crude site-specific melanoma incidence rates (unadjusted for other potential confounders, such as hair/eye color and genetic background due to data limitations, same for the rest of the paper when rate is mentioned) in the white population were calculated (Table 2) and are plotted in Figure 1A–D. As published by us before [4,5,18], white men showed an overall higher incidence rate (8.94 per 100,000 person-years in men vs. 2.64 in women) as compared to women in the Head/Neck region, and the trend remained significant after the age of 30 (Table 2, Figure 1A). In the Upper region, younger women (<55 years) and older men (≥55 years) showed higher rates than the opposite sex (Figure 1B). A similar pattern was observed in the Trunk region, where younger women (<40 years) and older men (≥40 years) showed greater rates compared to the opposite sex (Figure 1C). At the age of 40–84, the rates stayed at a relatively stable plateau in women and dropped slightly after the age of 80. By contrast, there was a continuous increase in men’s Trunk region with the age of the population. Similarly to elderly women, there was also a drop in the incidence rate at 85+ of age. Men exhibited higher rates in the Trunk region as compared to women, with an incidence rate ratio (IRR) of 0.45 (female to male). In the Lower region, a monotonously higher rate in women was observed as compared to men across all age groups (Table 2, Figure 1D). The rate in men also increased with the age of the population, but with a slope less steep than in women. Overall, the incidence rate ratio (female to male) was 2.18 in the Lower region.

**Figure 2:**
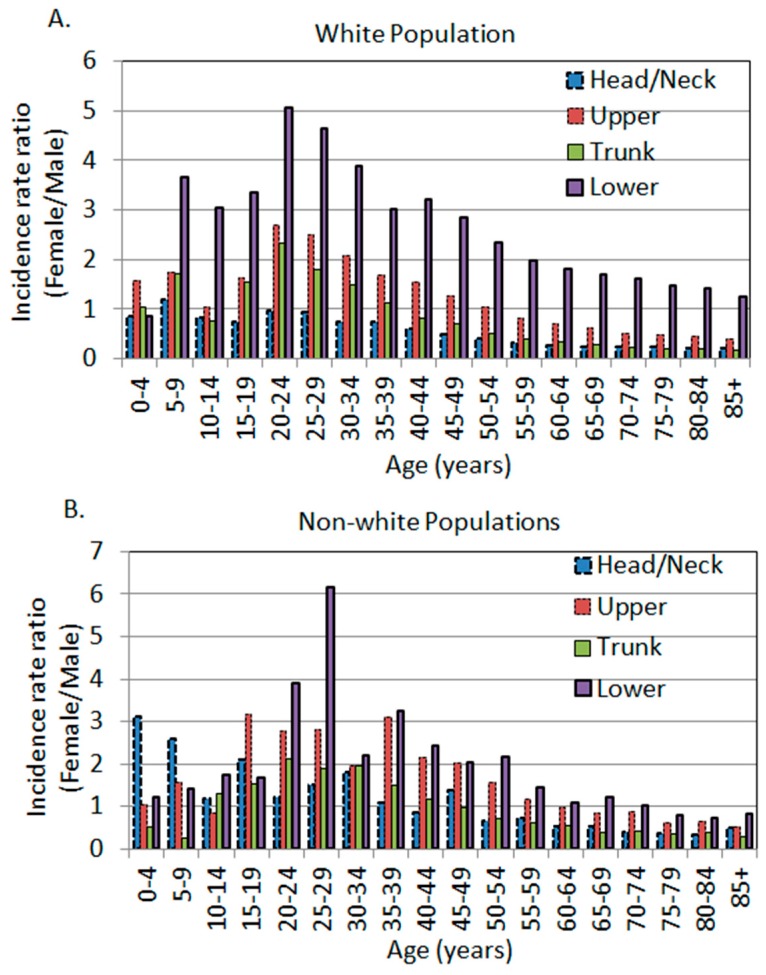
The age-specific female to male incidence rate ratios by anatomic site. (**A**) White population. (**B**) Non-white populations, including black Americans, Hispanics, American Indians/Alaska Natives, and Asian Americans/Pacific Islanders (SEER 18 data, 2000–2015).

When all body sites were combined, men’s incidence rate was higher than that of women. When stratifying the incidence rate by age, there was a uniformly spiked difference at the age of 20–24, with women exhibiting higher incidence rates (Figure 2A). In this age category, the female to male IRRs in the Upper, Trunk, and Lower regions were 2.69 (95% CI: 2.26, 3.21), 2.33 (95% CI: 2.07, 2.61), and 5.05 (95% CI: 4.20, 6.07), respectively. There was a non-significant surge at the age of 20–24 (IRR 0.96, 95% CI: 0.80, 1.14) in the Head/Neck region (Table 2, Figure 2A).

### 3.3. Sex Differences in Age- and Body Site-Specific Melanoma Incidence Rates in the Non-White Populations

The unadjusted age-, site-, and sex-specific incidence rates and female to male rate ratios in the non-white race groups were listed in Table 3. The rate ratios are plotted in Figure 1E–H. The Head/Neck region in the non-whites exhibited a similar pattern to other body sites where there was a significantly higher rate in women at younger ages. The spiked difference was observed at the age of 30–34 years with a female to male IRR of 1.81 (95% CI: 1.21, 2.72). The Upper body, Trunk, and Lower regions each exhibited a spiked difference at the age of 35–39 (IRR 3.10, 95% CI: 2.20, 4.36), 20–24 (IRR 2.12, 95% CI: 1.29, 3.46), and 25–29 (IRR 6.17, 95% CI: 3.63, 10.50), respectively (Table 3, bolded). Overall, non-white men also showed higher incidence rates in their Head/Neck, Trunk, and Lower body regions, just like Caucasian men. However, the magnitude of differences in non-whites seemed to be not as dramatic as in whites, as the IRRs were closer to 1 in non-whites in each body site and in all sites combined (Table 2 and Table 3, Figure 2).

### 3.4. The Age-Dependent Sex Differences Showed Younger Women and Older Men Were More Susceptible in Comparison to Their Opposite Sex of the Same Age

We and others have previously reported that the unadjusted age-specific female to male IRRs exhibited a spiked difference around the age of 20–24 years in both the white and non-white populations [4,26], with non-whites showing a slight shift of age from 20–39 years [26]. This trend was, again, observed in three out of four major body sites in whites, and all four body sites in non-whites (Figure 2A,B). The only outlier was the Head/Neck area in whites, which did not show a significant difference at 20–24 years of age, and the point estimate of IRR was close to 1 (Figure 2A). Regardless of the body sites, after the age of 20–24 years, the female to male IRR decreased with increasing age in the population in all body sites in the white population (Figure 2A). This trend remained the same in non-white populations but with some fluctuations and shifting of age (Figure 2B).

### 3.5. The Yearly Trend of Melanoma Incidence Rates in Each Body Site

**Figure 3:**
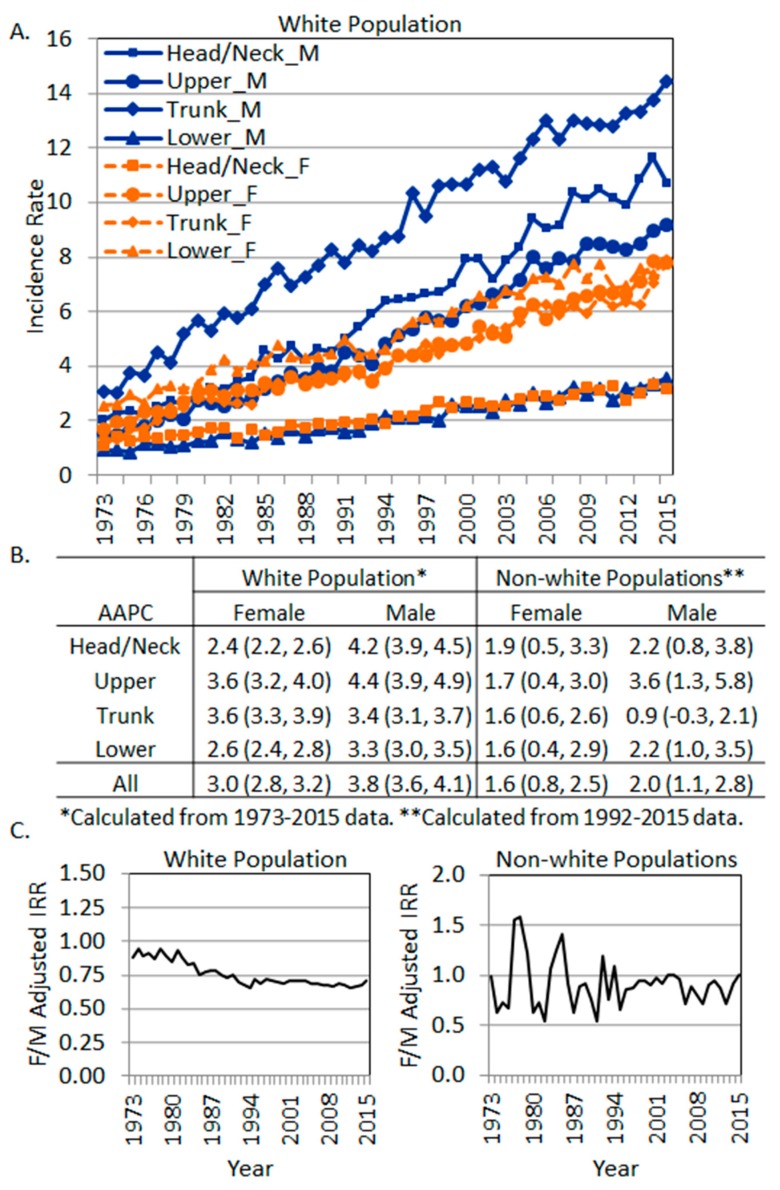
The trend of site-specific melanoma incidence rates by sex. (**A**) Incidence rates in the white population. (**B**) Average annual percentage change of site-specific incidence rates in each sex. (**C**) Overall female to male incidence rate ratios (SEER 9 data, 1973–2015). Left, white population. Right, non-white populations.

In order to examine whether each body site exhibited different annual percentage change in the age-adjusted incidence rates, we further retrieved SEER*Stat data from 1973 to 2015 for each of the four major body sites. The yearly trend of the incidence rates in the whites is plotted in Figure 3A, and the average annual percentage changes (AAPCs) were produced by the SEER Joinpoint Regression [28] Program. All AAPCs were significantly positive, indicating an increased trend of incidence rates over the period of 1973–2015. Interestingly, in the white population, the highest AAPC was in the shoulder and upper extremity region in men (AAPC 4.4, 95% CI: 3.9, 4.9), followed by the Head/Neck area in men (AAPC 4.2, 95% CI: 3.9, 4.5). The lowest AAPC was in women’s Head/Neck region (AAPC 2.4, 95% CI: 2.2, 2.6), followed by the hip and lower extremity region in women (AAPC 2.6, 95% CI: 2.4, 2.8). Overall, men exhibited higher AAPCs than women (3.8% vs. 3.0%) (Figure 3B). The overall annual female to male adjusted IRRs are plotted in Figure 3C (left panel). Apparently, there was a decreased trend of the female to male incidence rate ratios from 1973–1994, then the ratio stabilized at around 0.7 in the white population (Figure 3C, left panel).

In non-white populations, the AAPCs were produced using SEER*Stat data from 1992 to 2015 due to zero cases in some body sites from 1973–1991. AAPCs were significantly positive in all body sites in both genders except for men’s trunk, which showed a non-significant increase (AAPC 0.9, 95% CI: −0.3, 2.1). Overall, men showed a higher AAPC of increase than women. In non-white women, the AAPCs were similar among different body sites, but in men, the AAPCs varied from 1.6 (in the Lower region) to 3.6 (in the Upper region). The annual female to male adjusted IRRs in the non-white populations showed fluctuations around 1, with females showing slightly lower overall incidence rates over the years (Figure 3C, right panel).

## 4. Discussion

In this current study, we compared the gender differences between whites and non-whites in terms of their mean age of diagnosis, unadjusted age-specific body-site incidence rates, and AAPCs over the years. We first observed that the previously reported gender differences in the white population in different anatomic sites were confirmed in our data [8,9,10,16]. In other words, the most notable gender differences in melanoma incidence rates were in the head and neck region (higher in men) and hip and lower extremities region (higher in women). This was also observed in the non-white populations in the current study. Secondly, age-specific differences in each anatomic site were similar between the white and non-white populations, with females universally exhibiting higher rates at younger ages and males exhibiting higher rates at older ages, except for the head and neck region in whites. However, the mean age of diagnosis was significantly younger in the non-white populations than in whites. The gender differences in the mean age of diagnosis were significant among the body sites in whites but not in non-whites. The overall gender differences in the non-white populations were not as dramatic as in whites (female to male IRR was 0.63 in whites and 0.90 in non-whites, Table 2 and Table 3). Lastly, all body sites from both genders and all race groups showed positive AAPCs but with varying magnitudes. The shoulder and upper extremities showed the fastest increase in AAPCs in men in all race groups. Overall, whites showed a faster increase than non-whites.

In terms of the increase in the incidence rate of melanoma (AAPC) over the past 43 years (1973–2015), all race groups showed increased incidence rates, with whites showing a faster increase than non-whites overall and in each body site. It has been hypothesized that the increased ambient UV radiation as a result of depleted ozone levels might have caused the continued increase in melanoma incidence rates [29,30]. If this hypothesis had proven to be true, we would expect a higher AAPC in the sun-exposed body regions such as the head and neck areas. However, the fastest increase in men was observed in the current study in the Upper regions in all races, in the Trunk and Upper regions in white women, and in the Head/Neck region in non-white women. Therefore, an increase in the ambient UV dose cannot fully explain these observed phenomena. Increased ambient UV radiation seemed only able to explain the observed differences in the Head/Neck region, where white men showed a faster increase than white women, who usually use more cosmetics and sunscreen. Women’s long hair may also play a protective role. However, in both white men and women, the Lower region that was occasionally sun-exposed showed a slower increase in AAPC than the Trunk region which was often non-exposed. The ambient UV dose theory apparently could not be the best explanation for this observation.

Cancer is often a result of the interaction between the environment and genetics. Solar UV radiation is the most important risk factor for melanoma. We proposed a hypothesis that when UV radiation does not provide a good explanation, genetics and the related pathophysiological factors may play more important roles in melanoma transformation. This hypothesis is supported by the body site distribution of melanoma incidence rate as observed in the current study. The age-specific incidence rate ratios of gender and race-specific melanoma statistics also support this hypothesis as discussed in the following paragraphs.

For the unadjusted age-specific incidence rate ratios of gender, we showed in the current study again that there was a universally higher incidence rate in young women in all body sites and among all races. The ratio trends reversed after about 40 to 50 years of age, with men exhibiting higher rates at older ages. In experimental mice, a single dose of UV treated on newborns led to melanoma development after 6 months [31], suggesting that UV-induced mutations required a latent time for normal cells to progress to tumors. It is thus understandable that UV-induced melanomas are frequently diagnosed at an older age, as most of the UV radiation received during a lifetime is after the age of 18 [32]. In contrast, it is also perceivable that pathophysiological factors (largely genetics) may highly likely be associated with melanomas diagnosed at younger ages. We previously reported that the melanoma incidence rate was associated with geographical UV only in men [18], also supporting this hypothesis. If our hypothesis is proven true, the higher melanoma incidence rate in young women is perhaps not completely due to their UV behaviors, as widely assumed.

Race-specific melanoma statistics also support our hypothesis. Non-whites are less impacted by UV exposure due to their darker skin pigmentation, which provides additional UV protection [33]. However, the mean age of diagnosis of melanoma in non-whites was significantly younger than that of whites for both genders. The mean age of diagnosis was also much younger for trunk melanomas (less UV-exposed) than the head and neck melanomas (more UV-exposed) for both genders and all race groups.

On the hip and lower extremities, the overall age-adjusted IRRs were quite different among race groups (2.18 in the whites vs. 1.30 in the non-whites). However, at the age of 20–24 years, the magnitude of sex differences in whites was similar to that in non-whites (IRR 5.05 in whites, age 20–24 vs. 6.17 in non-whites, age 25–29), which represented the highest IRRs among all age groups and all body sites. If the higher rates in white women were attributed to clothing style-associated UV behaviors, then why did the relatively UV-protected non-white women show an even higher IRR on the lower extremities? Therefore, these results suggested that: (1) It is highly likely that the melanomas growing on the hip and lower extremities have an additional site-specific pathophysiological risk factor(s) and (2) melanomas growing at younger ages are less influenced by UV radiation.

Our results in the current study strongly suggested that melanomas developed at younger ages are attributed more to genetic and/or pathophysiological factors than the environmental factor, while later-onset melanomas are associated more with UV radiation. To the best of our knowledge, this hypothesis that melanomas exhibit an age-specific causal factor has not been explicitly proposed to date. Further evidence can also be found in the literature. For example, germline MC1R variants (loss of function) have been linked to melanomagenesis, and a major pathway is through altering melanin synthesis [34]. Many MC1R variants are known to be the “red hair” variants, because these individuals synthesize a large amount of pheomelanin which exacerbates their vulnerability to sun exposure [35]. In this case, both germline variants and UV exposure are responsible for the end result of melanoma. However, because of the research and public education of melanoma awareness, people carrying these variants may be more careful in avoiding sun exposure and in seeking dermatology screening, resulting in early diagnosis. Under these conditions, genetics, rather than UV exposure, may be more influential in earlier diagnosis. Interestingly, it was recently found that the red hair variants of MC1R differentially affected melanoma outcome between men and women [36], further supporting the gender differences in melanoma arising from a genetic background.

Nevertheless, there were a few limitations in the current study. First, the coarse categorization of melanomas by wide body area groupings in the SEER database limited our ability to differentiate more refined differences in tumor distributions between genders and among age strata. For example, we grouped the head and neck regions together but in fact, due to hair protection, scalp and face may receive different levels of UV exposure. Secondly, although the US SEER database provides population-based cancer information and personal UV exposure levels are well-documented at the individual level, there is no information on UV-associated behaviors, hairstyles, clothing, and hair-removal preferences that we could have used to sort out confounding effects and strengthen our hypothesis. Therefore, due to data limitations, our results could not control for these potential confounders and are considered to be unadjusted. Whenever possible, we used known confounders for our adjustment, such as age in calculating the overall incidence rates. Lastly, this type of data analyses was reported in similar studies in the white population by many others previously [9,10,16,23]. One strength of the current study was that we included different race groups in comparison to validate the observed tumor rates in different body areas, stratified by age, to generate our new hypothesis.

It is well documented that UV radiation and oxidative stress are linked [37], both of which can be differentially impacted by sex hormones. As redox biology is unique in melanocytes (evidenced by its regulation via melanocytic master regulator MiTF [38,39]), melanocytes may undergo a lineage-specific UV-induced DNA damage repair which is highly likely impacted by hormones and thus result in differential mutation rates. In fact, a gender disparity in mutation burden in melanoma samples from The Cancer Genome Atlas (TCGA) dataset was observed [40], providing additional evidence to our hypothesis.

## 5. Conclusions

In conclusion, our results showed various gender differences in age- and body site-specific melanoma incidence rates and the trend of incidence rates over the years. Based on these data, we postulated that melanomas at younger ages are less impacted by UV radiation, and a total net increase of solar UV radiation may not completely explain the various sex differences in melanoma tumor distribution in different body sites. Further research in gender-related and/or body site-specific pathophysiological factors as a unique determinant in cutaneous melanoma is warranted.

## Figures and Tables

**Table 1:** Cutaneous melanoma patient characteristics of the SEER18 database (2000–2015).

	Anatomic Site		Head/Neck	Upper	Trunk	Lower	All Sites
White	Number of cases (N, %)	Male	42,496 (16.2%)	36,560 (13.9%)	59,779 (22.8%)	13,831 (5.3%)	152,666 (58.2%)
		Female	15,534 (5.9%)	31,830 (12.1%)	29,017 (11.1%)	33,083 (12.6%)	109,464 (41.8%)
		Total	58,030 (22.1%)	68,390 (26.0%)	88,796 (33.9%)	46,914 (17.9%)	262,130 (100%)
	Mean age of diagnosis	Male	70	67	65	62	63
		Female	66	62	57	59	57
		*p*-value	**0.03** *	0.51	**0.02**	**0.01**	0.18
Non-white	Number of cases (N, %)	Male	1176 (10.4%)	955 (8.5%)	1572 (13.5%)	1385 (12.3%)	5088 (45.0%)
		Female	946 (8.4%)	1441 (12.8%)	1364 (12.1%)	2456 (21.7%)	6207 (55.0%)
		Total	2122 (18.8%)	2396 (21.2%)	2936 (26.0%)	3841 (34.0%)	11,295 (100%)
	Mean age of diagnosis	Male	63	57	59	60	59
		Female	57	50	53	55	54
		*p*-value	0.34	0.24	0.33	0.08	0.69
*p*-value (white vs. non-white, age of diagnosis)		Male	**0.0009**	**0.0002**	**0.0001**	**<0.0001**	**0.0002**
		Female	**0.0008**	**<0.0001**	**<0.0001**	**<0.0001**	**<0.0001**

* Bolded: *p* < 0.05.

**Table 2:** Age- and site-specific incidence rates (per 100,000 person-years) and rate ratios (F/M) in the white population.

	Head/Neck			Upper			Trunk			Lower			All Sites		
Age	F	M	Ratio (95% CI)	F	M	Ratio (95% CI)	F	M	Ratio (95% CI)	F	M	Ratio (95% CI)	F	M	Ratio (95% CI)
0–4	0.04	0.05	0.86 (0.43, 1.75)	0.02	0.01	1.57 (0.44, 5.57)	0	0	NA	0.03	0.03	0.87 (0.38, 2.02)	0.09	0.10	0.96 (0.59, 1.55)
5–9	0.05	0.04	1.20 (0.59, 2.46)	0.06	0.03	1.75 (0.86, 3.58)	0.05	0.03	1.72 (0.81, 3.64)	0.08	0.02	3.67 (1.67, 8.06)	0.25	0.13	1.91 (1.33, 2.75)
10–14	0.11	0.13	0.83 (0.54, 1.27)	0.08	0.08	1.05 (0.63, 1.76)	0.10	0.13	0.75 (0.48, 1.18)	0.14	0.05	3.03 (1.74, 5.26)	0.43	0.39	1.12 (0.89, 1.41)
15–19	0.28	0.38	0.74 (0.57, 0.96)	0.34	0.21	1.63 (1.22, 2.17)	0.84	0.55	1.53 (1.28, 1.84)	0.54	0.16	3.34 (2.49, 4.48)	2.00	1.30	1.54 (1.37, 1.74)
20–24	0.70	0.73	0.96 (0.80, 1.14)	**1.29**	**0.48**	**2.69** (2.26, 3.21) **	**2.71**	**1.16**	**2.33** **(2.07, 2.61)**	**1.89**	**0.37**	**5.05** **(4.20, 6.07)**	**6.59**	**2.75**	**2.40** **(2.23, 2.58)**
25–29	1.14	1.22	0.93 (0.81, 1.07)	2.31	0.92	2.50 (2.20, 2.85)	4.44	2.46	1.81 (1.66, 1.96)	3.45	0.75	4.63 (2.06, 5.29)	11.33	5.35	2.12 (2.01, 2.24)
30–34	1.26	1.67	0.75 (0.67, 0.85)	3.40	1.65	2.07 (1.87, 2.28)	5.57	3.78	1.48 (1.38, 1.58)	4.98	1.28	3.87 (3.49, 4.29)	15.20	8.37	1.82 (1.74, 1.90)
35–39	1.54	2.09	0.74 (0.66, 0.83)	4.27	2.53	1.69 (1.55, 1.83)	6.48	5.77	1.12 (1.06, 1.19)	6.30	2.08	3.02 (2.78, 3.28)	18.60	12.48	1.49 (1.43, 1.55)
40–44	1.81	3.01	0.60 (0.55, 0.66)	5.86	3.81	1.54 (1.44, 1.64)	7.13	8.72	0.82 (0.78, 0.86)	8.10	2.52	3.21 (2.99, 3.46)	22.90	18.06	1.27 (1.23, 1.31)
45–49	2.23	4.51	0.49 (0.45, 0.54)	7.54	6.02	1.25 (1.18, 1.32)	8.52	12.41	0.69 (0.66, 0.72)	9.50	3.34	2.84 (2.67, 3.03)	27.78	26.29	1.06 (1.03, 1.09)
50–54	2.83	6.94	0.41 (0.38, 0.44)	9.17	8.85	1.04 (0.99, 1.09)	9.06	17.61	0.51 (0.49, 0.54)	10.25	4.36	2.35 (2.22, 2.50)	31.32	37.76	0.83 (0.81, 0.85)
55–59	3.54	11.13	0.32 (0.30, 0.34)	10.72	13.03	0.82 (0.79, 0.86)	9.39	24.11	0.39 (0.37, 0.41)	10.65	5.34	1.99 (1.88, 2.12)	34.29	53.62	0.64 (0.62, 0.66)
60–65	4.72	17.85	0.26 (0.25, 0.28)	12.94	18.50	0.70 (0.67, 0.73)	10.06	30.79	0.33 (0.31, 0.34)	11.62	6.40	1.81 (1.71, 1.93)	39.34	73.54	0.53 (0.52, 0.55)
65–69	7.03	27.78	0.25 (0.24, 0.27)	15.29	24.72	0.62 (0.59, 0.65)	10.69	39.82	0.27 (0.26, 0.28)	13.11	7.70	1.70 (1.60, 1.81)	46.12	100.02	0.46 (0.45, 0.47)
70-74	9.56	39.87	0.24 (0.23, 0.25)	16.17	32.14	0.50 (0.48, 0.53)	10.40	44.49	0.23 (0.22, 0.25)	13.69	8.47	1.62 (1.51, 1.73)	49.82	124.99	0.40 (0.39, 0.41)
75–79	12.08	52.09	0.23 (0.22, 0.24)	18.13	37.71	0.48 (0.46, 0.51)	10.23	49.77	0.21 (0.19, 0.22)	14.55	9.95	1.46 (1.36, 1.57)	54.98	149.52	0.37 (0.36, 0.38)
80–84	15.11	69.56	0.22 (0.21, 0.23)	18.62	42.05	0.44 (0.42, 0.47)	9.43	50.44	0.19 (0.17, 0.20)	14.64	10.38	1.41 (1.30, 1.53)	57.80	172.44	0.34 (0.33, 0.35)
85+	19.03	89.10	0.21 (0.20, 0.22)	17.01	43.47	0.39 (0.37, 0.41)	7.46	45.08	0.17 (0.15, 0.18)	13.72	11.01	1.25 (1.14, 1.36)	57.22	188.65	0.30 (0.29, 0.31)
All age *	2.64	8.94	0.30 (0.27, 0.32)	5.60	7.40	0.76 (0.71, 0.80)	5.33	11.82	0.45 (0.43, 0.48)	5.98	2.74	2.18 (2.02, 2.36)	19.55	30.90	0.63 (0.61, 0.65)

* Age-adjusted. ** Bolded: women exhibited the highest incidence rate ratios with statistical significance (*p* < 0.05).

**Table 3:** Age- and site-specific incidence rates (per 100,000 person-years) and rate ratios (F/M) in the non-white populations.

	Head/Neck			Upper			Trunk			Lower			All Sites		
Age	F	M	Ratio (95% CI)	F	M	Ratio (95% CI)	F	M	Ratio (95% CI)	F	M	Ratio (95% CI)	F	M	Ratio (95% CI)
0–4	0.012	0.004	3.12 (0.32, 30.0)	0.012	0.011	1.04 (0.21, 5.16)	0.004	0.007	0.52 (0.05, 5.74)	0.027	0.022	1.21 (0.41, 3.61)	0.054	0.045	1.21 (0.56, 2.62)
5–9	0.020	0.008	2.60 (0.50, 13.4)	0.012	0.008	1.56 (0.26, 9.32)	0.004	0.015	0.26 (0.03, 2.32)	0.044	0.030	1.43 (0.57, 3.55)	0.079	0.061	1.30 (0.67, 2.51)
10–14	0.031	0.026	1.19 (0.43, 3.28)	0.016	0.019	0.83 (0.22, 3.10)	0.02	0.015	1.30 (0.35, 4.84)	0.059	0.034	1.73 (0.76, 3.96)	0.126	0.094	1.33 (0.79, 2.25)
15–19	0.040	0.019	2.12 (0.73, 6.21)	0.036	0.011	3.18 (0.86, 11.8)	0.064	0.042	1.54 (0.72, 3.33)	0.076	0.046	1.68 (0.82, 3.46)	0.217	0.118	1.85 (1.19, 2.88)
20–24	0.073	0.060	1.22 (0.62, 2.38)	0.146	0.053	2.78(1.50, 5.15)	**0.191**	**0.090**	**2.12** **(1.29, 3.46)**	0.191	0.049	3.91 (2.11, 7.22)	0.602	0.252	2.39 (1.79, 3.19)
25–29	0.172	0.114	1.50 (0.94, 2.41)	0.188	0.067	2.81 (1.61, 4.90)	0.274	0.146	1.88 (1.26, 2.81)	**0.388**	**0.063**	**6.17** **(3.63, 10.5)**	**1.022**	**0.390**	**2.62** **(2.08, 3.31)**
30–34	**0.268**	**0.148**	**1.81** **(1.21, 2.72)** **	0.404	0.206	1.97 (1.40, 2.76)	0.528	0.272	1.95 (1.45, 2.62)	0.536	0.243	2.21 (1.62, 3.00)	1.737	0.868	2.00 (1.70, 2.36)
35–39	0.234	0.213	1.10 (0.75, 1.61)	**0.579**	**0.187**	**3.10** **(2.20, 4.36)**	0.63	0.417	1.51 (1.17, 1.95)	0.817	0.252	3.24 (2.42, 4.35)	2.259	1.069	2.11 (1.82, 2.46)
40–44	0.264	0.306	0.86 (0.61, 1.23)	0.716	0.334	2.14 (1.62, 2.83)	0.649	0.552	1.18 (0.92, 1.50)	0.945	0.39	2.42 (1.88, 3.12)	2.575	1.583	1.63 (1.42, 1.86)
45–49	0.400	0.287	1.39 (0.99, 1.96)	0.677	0.334	2.03 (1.51, 2.73)	0.732	0.757	0.97 (0.77, 1.22)	1.098	0.538	2.04 (1.62, 2.58)	2.907	1.916	1.52 (1.33, 1.73)
50–54	0.368	0.560	0.66 (0.48, 0.90)	0.974	0.628	1.55 (1.21, 1.98)	0.832	1.181	0.70 (0.57, 0.87)	1.489	0.689	2.16 (1.73, 2.70)	3.664	3.058	1.20 (1.07, 1.35)
55–59	0.603	0.838	0.72 (0.54, 0.96)	0.841	0.728	1.16 (0.75, 1.23)	0.806	1.297	0.62 (0.49, 0.79)	1.535	1.06	1.45 (1.17, 1.80)	3.785	3.923	0.96 (0.85, 1.09)
60–65	0.579	1.048	0.55 (0.40, 0.76)	1.214	1.265	0.96 (0.75, 1.23)	1.049	1.892	0.55 (0.44, 0.70)	1.959	1.816	1.08 (0.88, 1.32)	4.801	6.021	0.80 (0.71, 0.90)
65–69	0.927	1.733	0.54 (0.40, 0.72)	1.171	1.401	0.84 (0.63, 1.11)	0.988	2.531	0.39 (0.30, 0.51)	2.635	2.154	1.22 (0.99, 1.51)	5.722	7.819	0.73 (0.65, 0.83)
70–74	1.199	3.085	0.39 (0.29, 0.51)	1.678	1.923	0.87 (0.66, 1.16)	1.151	2.832	0.41 (0.31, 0.54)	2.861	2.789	1.03 (0.82, 1.28)	6.890	10.63	0.65 (0.57, 0.74)
75–79	1.597	4.188	0.38 (0.29, 0.50)	1.492	2.35	0.63 (0.46, 0.88)	1.156	3.103	0.37 (0.27, 0.52)	3.404	4.308	0.79 (0.63, 0.99)	7.647	13.95	0.55 (0.48, 0.63)
80–84	1.812	5.539	0.33 (0.24, 0.45)	1.873	2.842	0.66 (0.46, 0.94)	1.359	3.613	0.38 (0.26, 0.54)	3.715	5.058	0.73 (0.57, 0.95)	8.760	17.05	0.51 (0.44, 0.60)
85+	3.279	6.564	0.50 (0.38, 0.66)	1.689	3.25	0.52 (0.35, 0.77)	0.96	3.445	0.28 (0.18, 0.44)	4.372	5.20	0.84 (0.64, 1.11)	10.30	18.46	0.56 (0.48, 0.66)
All age *	0.383	0.676	0.57 (0.39, 0.82)	0.553	0.49	1.13 (0.80, 1.58)	0.504	0.763	0.66 (0.49, 0.90)	0.964	0.74	1.30 (1.00, 1.70)	2.403	2.669	0.90 (0.77, 1.05)

* Age-adjusted. ** Bolded: women exhibited the highest incidence rate ratios with statistical significance (*p* < 0.05).
